# Regulatory effect of anti-gp130 functional mAb on IL-6 mediated RANKL and Wnt5a expression through JAK-STAT3 signaling pathway in FLS

**DOI:** 10.18632/oncotarget.23917

**Published:** 2018-01-04

**Authors:** Ping Miao, Xiao Wei Zhou, Ping Wang, Rong Zhao, Ninan Chen, Chao Ying Hu, Xue Hua Chen, Liu Qian, Qi Wen Yu, Ji Ying Zhang, Rong Xu, Dong Yi He, Lian Bo Xiao, Pu Li, Mason Lu, Dong Qing Zhang

**Affiliations:** ^1^ Department of Laboratory Medicine, Renji Hospital, School of Medicine, Shanghai Jiao Tong University, Shanghai, China; ^2^ Reproductive Medical Center of Ruijin Hospital, School of Medicine, Shanghai Jiao Tong University, Shanghai, China; ^3^ Shanghai Jiao Tong University School of Medicine, XinHua Hospital, Shanghai, China; ^4^ Shanghai Institute of Immunology, Shanghai Jiao Tong University School of Medicine, Shanghai, China; ^5^ Department of Neurology, Shanghai Ninth People’s Hospital, Shanghai Jiao Tong University School of Medicine, Shanghai, China; ^6^ Department of Pediatrics, Ruijin Hospital and Ruijin Hospital North, Shanghai Jiao Tong University School of Medicine, Shanghai, China; ^7^ Central laboratory, Shanghai Xuhui Central Hospital, Shanghai, China; ^8^ Shanghai Guanghua Hospital of Integrated Traditional Chinese and Western Medicine, Shanghai, China; ^9^ MedAbome, Inc, Fremont, CA, USA

**Keywords:** rheumatoid arthritis, gp130, collagen II antibody-induced arthritis, receptor activator of nuclear factor κB ligand, STAT3

## Abstract

We investigated the effect on rheumatoid arthritis (RA) of an anti-gp130 monoclonal antibody (mAb) and its mechanism using RA fibroblast-like synoviocytes (FLS) and a collagen antibody–induced arthritis (CAIA) mouse model. We determined the interleukin 6 (IL-6), IL-6 receptor α (IL-6Rα), gp130, receptor activator of nuclear factor κB ligand (RANKL), matrix metalloproteinase 3 (MMP3), TIMP metallopeptidase inhibitor 1 (TIMP1), and Bcl-2 levels in RA and osteoarthritis (OA) serum and synovial fluid. RA FLS were cultured with or without IL-6/IL-6Rα; WNT5A and RANKL levels were detected. We generated an anti-gp130 mAb (M10) with higher affinity and specificity, blocked IL-6 signaling with it, and assessed its effects on the CAIA model, *WNT5A* and *RANKL* expression, and signal transducer and activator of transcription 3 (STAT3) phosphorylation. The IL-6 signaling system in patients with RA was increased; RANKL, MMP3, TIMP1, and Bcl-2 in RA bone were elevated. IL-6/IL-6Rα increased RA FLS *WNT5A* and *RANKL* expression. M10 ameliorated arthritis in the CAIA model, and inhibited RANKL, WNT5A, and Bcl-2 expression in RA FLS by blocking IL-6 signaling, likely via Janus kinase–STAT3 pathway downregulation. The IL-6–soluble IL-6Rα–gp130 complex is hyperactive in RA and OA. M10 may be the basis for a novel RA treatment drug.

## INTRODUCTION

Rheumatoid arthritis (RA) is a chronic systemic autoimmune disease which is characterized by the invasion and proliferation of synoviocytes and resulting in bone and cartilage destruction [1]. The exact cause of RA is unknown, but previous studies have shown that abnormal secretion of various cytokines by RA fibroblast-like synoviocytes (FLS) play key roles in its pathogenesis [2]. Inflammatory stimuli, especially IL-6, TNFα and IL-1, are potent FLS activators and can induce FLS to produce other cytokines, which in turn promotes autoimmunity, maintains chronic inflammatory synovitis, and drives bone and joint destruction [1].

IL-6 interacts with two receptors, an 80 kDa IL-6Rα(also known as gp80) and the ubiquitously expressed signal-transducing co-receptor molecule gp130(also know as IL-6Rβ), which thereupon form a hexameric signaling complex. The combination of IL-6/IL-6R and gp130 subsequently activates the Janus kinase (JAK) signalling pathway, which preferentially induces tyrosine phosphorylation of STAT3. The membrane -bound gp80 only present on certain leukocyte subsets and hepatocytes, while soluble gp80-mediated trans-signaling enables IL-6–driven stimulation of cells that do not express gp80, thereby expanding the responding range of cell types [2, 3]. IL-6 contributes to many local and systemic signs and features of RA, such as joint inflammation, joint damage, and induction of autoimmune process [4]. Local bone destruction is a characteristic of RA. Receptor activator of nuclear factor κB ligand (RANKL) is a critical component in RA bone erosion, enhancing osteoclast formation, function, and survival. FLS is the main source of RANKL in the inflamed joints of RA. RANKL mediated effects through binding to RANK on osteoclasts and their precursors surface [5, 6]. Factors such as IL-6, TNFα, IL-1, and IL-17 regulate RANKL expression and synthesis. Hashizume et al. demonstrated that IL-6 can stimulate RANKL expression in FLS associated with soluble IL-6R (sIL-6R) [7]. This suggests that IL-6 trans-signaling pathway might enhance osteoclastogenesis through elevated RANKL expression in the FLS of RA patients.

Besides inducing RANKL expression, the IL-6–gp130 system can regulate the expression of the apoptosis-related gene BCL2 through the JAK-STAT signaling pathway, resulting in excessive proliferation of synovial tissue, promoting RA occurrence and development.

Recently, several reports have indicated that WNT5A, a member of the noncanonical WNT5A family, is a conserved target of the STAT3 signaling cascade and may have important function in the pathogenesis of rheumatic diseases [8–10]. In fact, WNT5A is expressed in the synovial tissue of RA patients, and its expression is increased in RA FLS; WNT5A acts on osteoclast precursors and enhances the expression of RANK, thus promoting RANKL-induced osteoclastogenesis [10].

The goal of RA treatment is remission or low disease activity, ultimately slowing or preventing the progression of joint destruction. In RA and other inflammatory diseases, there are more than one million patients received treatment of TNF-α neutralizing agents, but about 45% patients with inflammatory arthritis are ineffective against TNF-α treatment. New drugs still needed to be developed. However, the harvest of IL-6–targeting therapy underlines the importance of blocking IL-6 in RA pathology.

Formulations targeting IL-6 cytokines are being studied extensively, such as the IL-6R blocker tocilizumab and selective small molecule inhibitors of IL-6. These patients failure to anti TNF-α treatment are effective using TCZ, so TNF-α and IL-6 play different roles in inflammation in joints. TCZ binds to both two forms of human IL-6R to inhibit IL-6 function, thereby reducing synovitis, cartilage and bone damage, and systemic inflammation in RA. Nevertheless, adverse effects have been reported in patients after TCZ treatment. The most common adverse events are infection, rash, biochemical abnormalities, and so on [11]. So it is more important to develop new selective and specific inhibition.

In the present study, we report our development of a novel antibody that binds to gp130 with high affinity, evaluate its effectiveness, and explore its possible mechanism of action. We describe the development, characterization, and *in vitro* and *vivo* performance of the anti-gp130 antibody. We present our findings on RANKL and WNT5A function in osteoclastogenesis and study the effect of the anti-gp130 antibody on RANKL and WNT5A in IL-6–stimulated RA FLS *in vitro* and in a collagen antibody–induced arthritis (CAIA) mouse model. Taken together, our findings support the anti-gp130 antibody as an important regulator of cytokine-induced RANKL and WNT5A expression and the development of bone erosion in RA.

## RESULTS

### Upregulation of IL-6/sIL-6Rα/gp130 and bone destruction–related factors in RA

**Figure 1:**
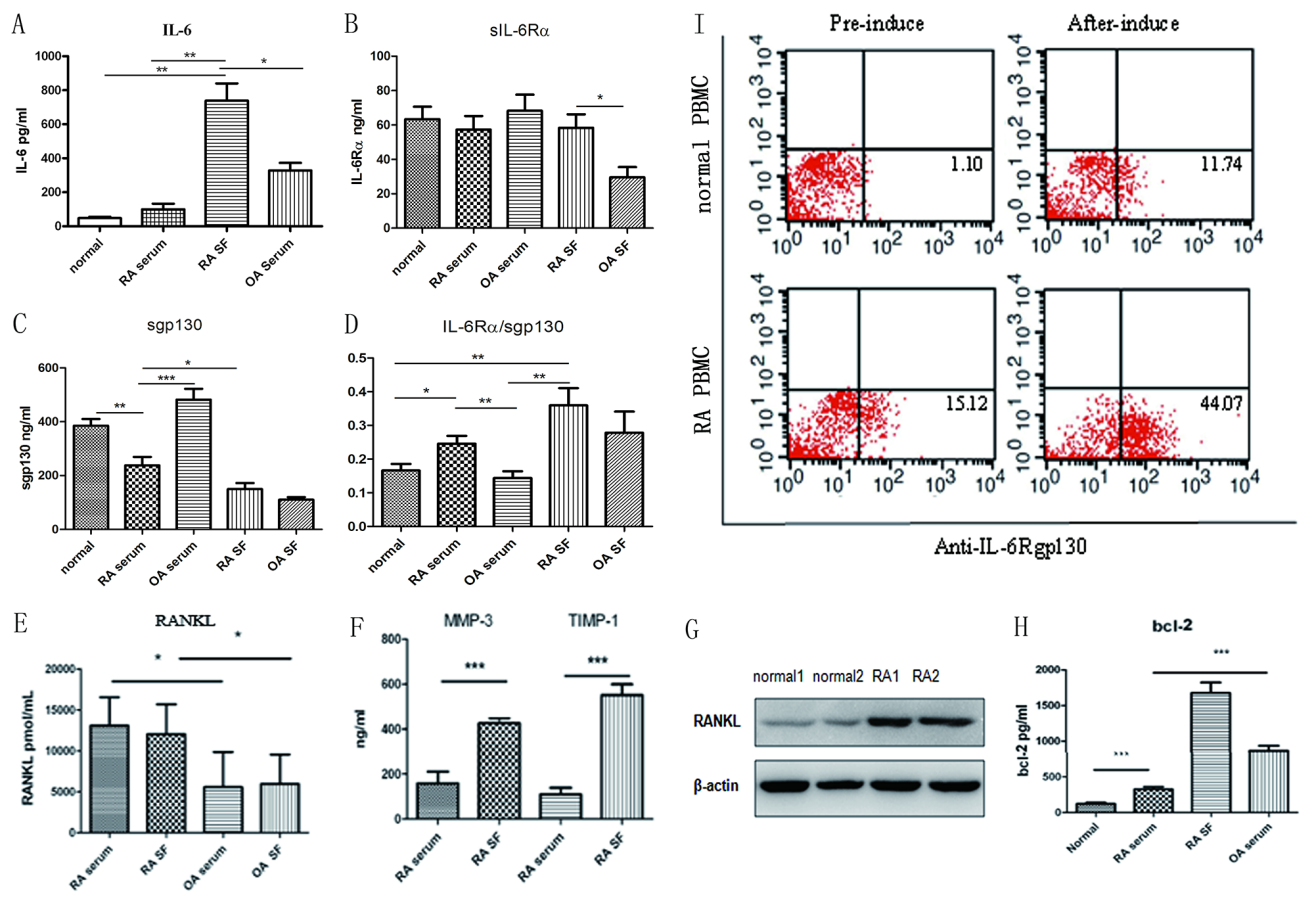
Analysis of IL-6/IL-6Rα/gp130 expression levels and RANKL in patients with RA **(A)** IL-6 levels in RA SF and in the control, OA, and RA serum. **(B, C)** sIL-6Rα and sgp130 levels in the control, OA, and RA serum and in the OA and RA SF. **(D)** Analysis of the sIL-6Rα and sgp130 ratio. **(E)** ELISA determination of soluble RANKL concentrations in RA and OA serum and SF. **(F)** Detection of MMP3 and TIMP1 in RA or OA serum and SF. **(G)** Western blot detection of RANKL expression in the bone tissues of patients with RA and the controls. **(H)** Bcl-2 levels in RA SF and in control, OA, and RA serum. **(I)** gp130 expression in the cells from the controls and patients with RA after 10-day induction with RANKL and GM-CSF; the percentage of gp130-positive cells was detected.

ELISA was used to compare the level of soluble inflammatory factors in the serum and synovial fluid (SF) of patients with RA with that in patients with osteoarthritis (OA) and in healthy controls. IL-6 was significantly elevated in RA SF compared to RA serum (*P* < 0.05), also was higher compared to OA serum (Figure 1A) (*P* < 0.01). Moreover, RA SF had increased sIL-6Rα levels compared to that of OA; these findings suggest that RA SF is more sensitive to IL-6 trans-signaling (Figure 1B) (*P* < 0.01). However, RA serum had the lowest expression of the IL-6 trans-signaling inhibitor sgp130 compared to OA serum and control serum. Therefore, the patients with RA had higher proportions of sIL-6Rα/sgp130, especially in SF (Figure 1C, 1D; control, *n* = 8; OA, *n* = 6; RA, *n* = 6). These findings indicate that the IL-6 signaling system is increased in patients with RA, as sgp130 is a natural antagonist of the gp130 signaling system in RA.

RA eventually leads to bone and cartilage destruction; osteoclasts play an important role in bone destruction. RANKL is critically involved in RA bone erosion by enhancing osteoclast formation, function, and survival. RANKL levels were significantly higher in RA serum and SF compared to that in OA serum and SF (Figure 1E; OA, *n* = 6; RA, *n* = 15; *P* < 0.01and *P* < 0.05, respectively). And western blotting showed high RANKL expression in RA bone tissues as compared to the controls (Figure 1G). RA SF had significantly higher levels of the bone destruction–related factors matrix metalloproteinase 3 (MMP3) and tissue inhibitor of metalloproteinases 1(TIMP1) than RA serum (Figure 1F, *P* < 0.001), suggesting that excessive immune response mediates the severe bone destruction and lesions in RA. The anti-apoptotic factor Bcl-2 also showed the same secretion pattern, being significantly increased in the OA and RA serum, and more so in RA SF, suggesting the excessive proliferation of synovial tissue (Figure 1H). To clarify the relevance of the IL-6–gp130 signaling system to RA, we detected gp130 expression in healthy controls and in patients with RA. Compared with the controls, the patients had higher gp130 expression, after 10-day induction by RANKL and GM-CSF; almost 60% of cells were osteoclasts, and the percentage of gp130-positive cells was significantly increased post-induction compared with pre-induction in both the control and RA groups (Figure 1I).

Then, we wondered whether blocking the IL-6–gp130 signaling pathway can affect the RA disease model and inhibit bone destruction. Due to their serious adverse effects, clinical application of the current IL-6– and IL-6Rα–targeting drugs is limited. To this end, we developed a functional mAb against gp130 and identified its biological characteristics and functions.

### Generation and characterization of anti-gp130 mAb

**Figure 2:**
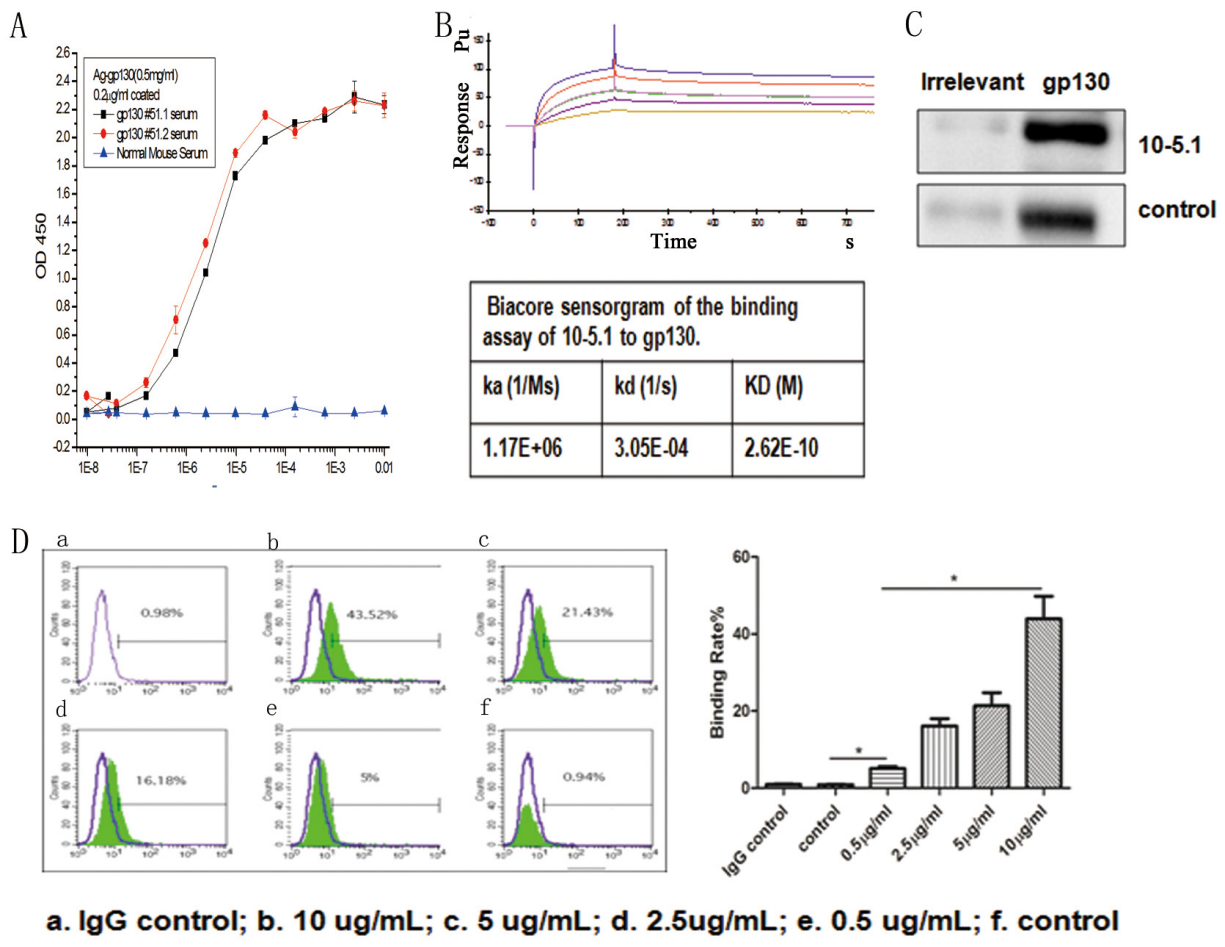
The characterization and binding effects of anti-gp130 mAb **(A)** Titration analysis of serum from gp130 immunized mice and binding to gp130 antigen. **(B)** Biacore sensorgram of the binding assay of M10 to gp130. **(C)** Western blot detection of gp130 expression in U266 cells using M10 and control anti gp130 mAb from R&D. **(D)** FACS detection of the binding characteristics of M10 to gp130 antigen by different doses. a. IgG control; b. 10 ug/ml; c. 5ug/ml; d.2.5ug/ml. e. control anti gp130 mAb from R&D.

We immunized 10 A/J mice with gp130 antigen. Before the final boost, we detected the serum-specific antibody titers of each immunized mouse. The serum from all mice had high binding signals to the gp130 antigen (Figure 2A). Following antibody hybridoma generation via the fusion of spleen cells from the immunized mice with Sp2/0 cells, high-throughput screening identified the hybridoma with the highest binding signal (M10), which was selected for subcloning and antibody affinity purification for further study. The M10 heavy chain belongs to immunoglobulin G 2b; the light chain is a kappa chain. Sequencing of the Fab fragments of the antibody variable region (light chain and heavy chain) revealed that the unique antigen connection region CDR (complementarity-determining region) was present in the variable region (data not shown). M10 bound human gp130 with an affinity of 2.62E-10 in a Biacore assessment involving captured antibody and soluble gp130 (Figure 2B). We used the human myeloma cell line U266 (which has high gp130 expression) to determine the binding capacity of M10. Western blotting showed a specific band of the expected 130-KD size; flow cytometry suggested that M10 and cell surface gp130 binding occurred in a dose-dependent manner (Figure 2C, 2D).

### M10 may alleviate arthritis severity in CAIA mice

**Figure 3:**
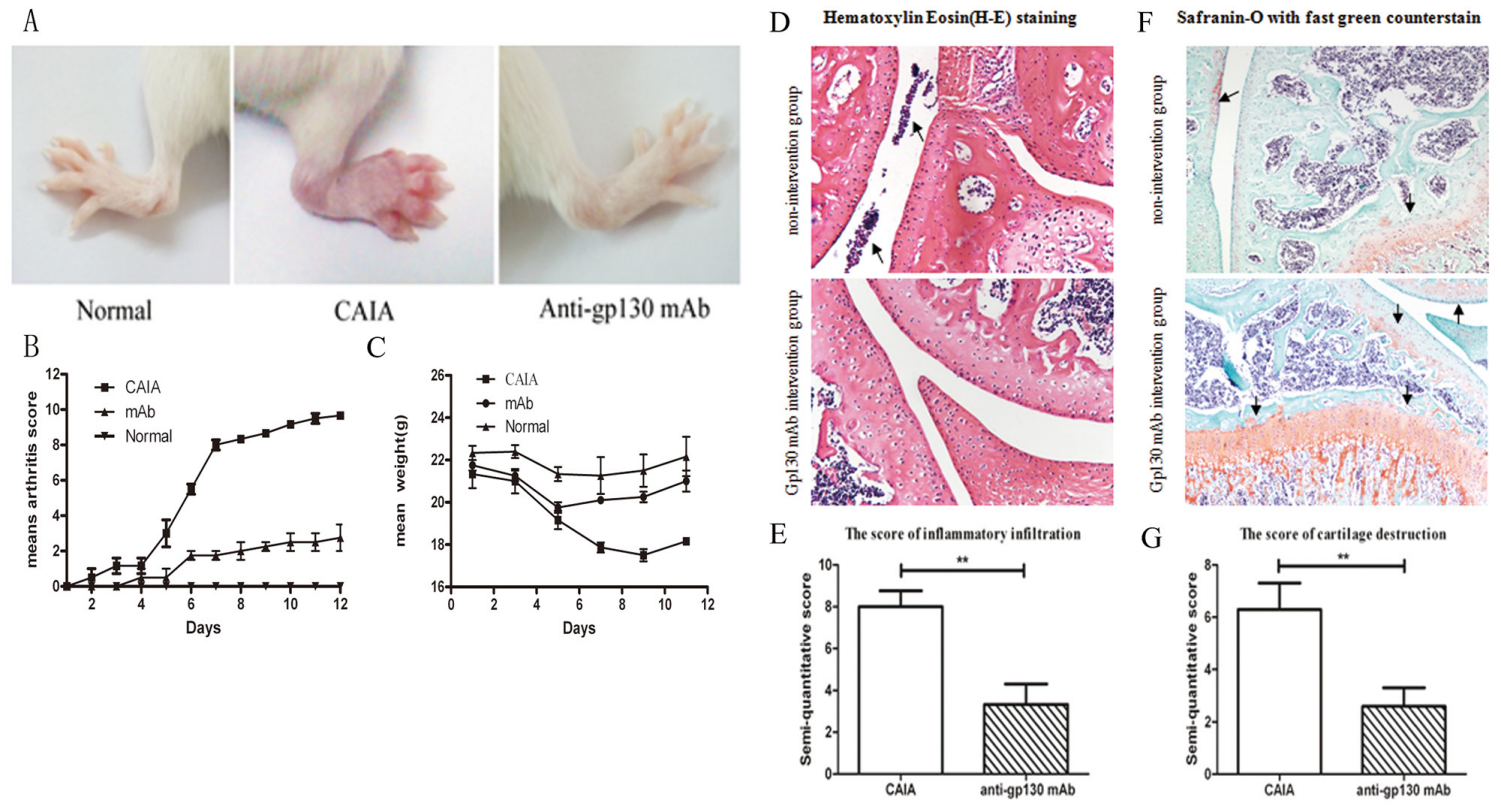
The effect of anti-gp130 mAb on CAIA mouse model **(A)** Photographs of representative hind-paws. **(B)** Arthritis scores. **(C)** Mean weights. **(D)** HE staining of paws and knees. **(E)** The score for infiltrated inflammatory cells in the articular cavity of the treated and untreated groups. **(F)** Safranin O staining with fast green counterstaining of paws and knees. **(G)** The cartilage destruction score in the treated and untreated groups.

In order to assess the *in vivo* efficacy of M10, we created a CAIA mouse model as described previously [12]. CAIA mice model is believed to imitate many factors of the human effector phase of RA. M10 was administered intraperitoneally on day 4. Total CAIA mice developed arthritis, and the clinical score increased on day 12 by degrees. CAIA mice that received M10 had alleviated CAIA signs and symptoms and decreased arthritis scores compared to the control group (Figure 3A–3C). Hematoxylin–eosin (HE) staining of the mouse paws and knees revealed decreased infiltrated inflammatory cells within the articular cavity after M10 treatment compared with the non-intervention group; also safranin O staining with fast green counterstaining underlines alleviated cartilage destruction in the intervention group (Figure 3D–3G).

These results indicate that M10 can potently attenuate the signs of arthritis *in vivo*. Accordingly, we wondered by what mechanism M10 interfered with RA progression significantly.

### M10 regulation of RANKL and WNT5A expression in IL-6/sIL-6Rα–stimulated RA FLS

**Figure 4:**
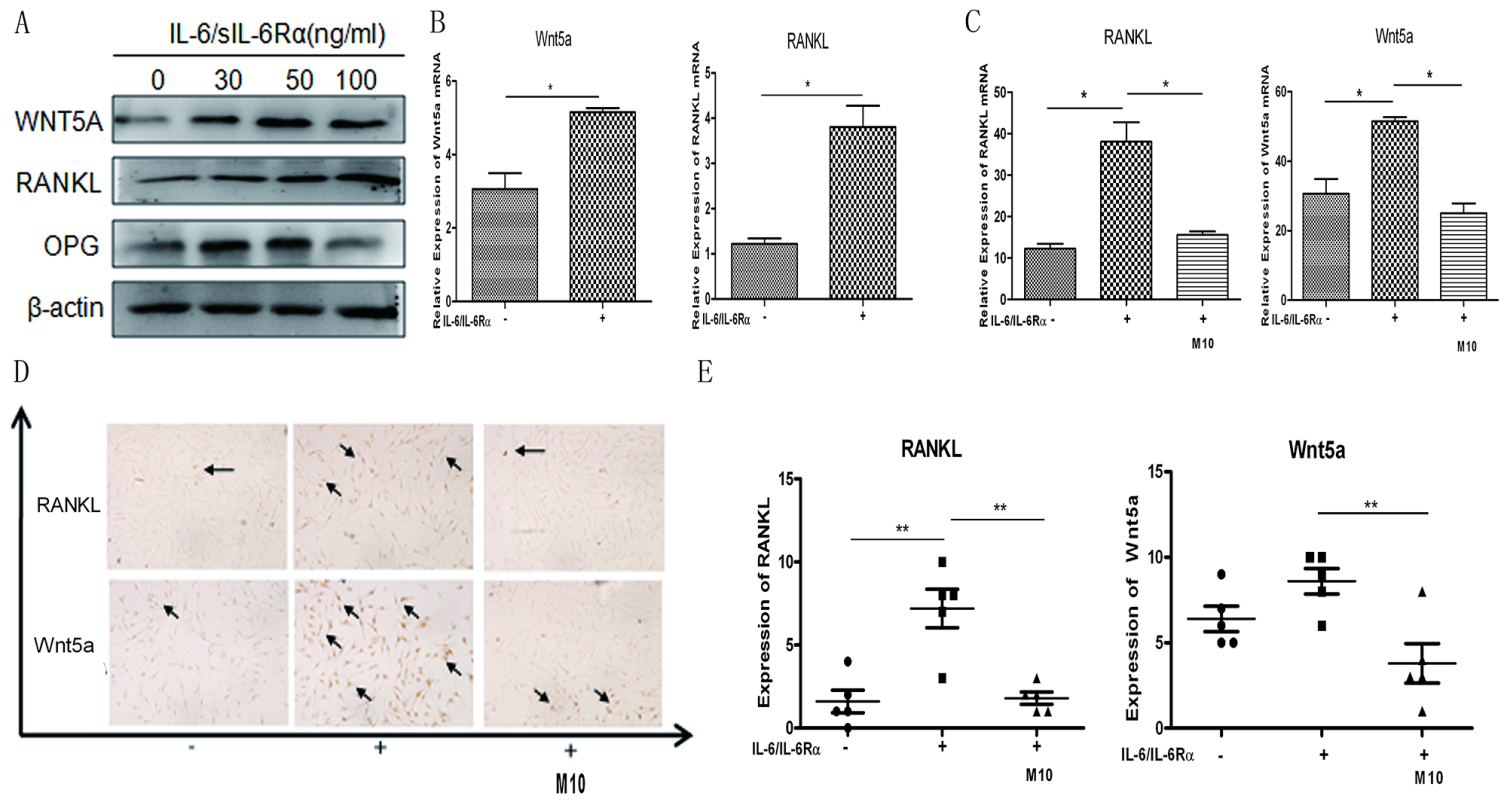
Changes in IL-6/sIL-6Rα–induced RANKL and WNT5A expression after M10 pretreatment of RA FLS **(A)** Western blot detection of WNT5A and RANKL expression in RA FLS following 3-day stimulation with IL-6 and sIL-6Rα. **(B)** Real-time PCR determination of *RANKL* and *WNT5A* mRNA in RA FLS following 3-day stimulation with IL-6 and sIL-6Rα. **(C)** Real-time PCR determination of *RANKL* and *WNT5A* mRNA in RA FLS pretreated with M10, and then cultured with IL-6/sIL-6Rα for 72 h. Data are normalized to β-actin and reported in relative expression units. **(D)** Immunostaining of RA FLS pretreated with M10 and cultured with IL-6/sIL-6Rα for 72 h (×200 magnification). Figures are representative of three independent experiments. **(E)** The number of RANKL- and WNT5A-positive staining cells.

RANKL and WNT5A are critical components in RA bone erosion. We evaluated whether IL-6 stimulates increased RANKL and WNT5A expression in RA FLS, thereby participating in RA osteoclastogenesis. RA FLS were stimulated with different concentrations of IL-6/sIL-6Rα for three days; subsequently, RANKL and WNT5A were upregulated in tandem with the IL-6/sIL-6Rα concentrations (Figure 4A). The results were confirmed at mRNA level (Figure 4B). M10 markedly downregulated *RANKL* and *WNT5A* mRNA expression in RA FLS induced with 50 μg/mL IL-6/sIL-6Rα (both, *P* < 0.001) (Figure 4C). Immunohistochemistry confirmed the results (Figure 4D). M10 obviously downregulated the amount of RANKL- and WNT5A-stained cells relative to non-induced RA FLS cultured under the equal experiment conditions (Figure 4E).

### M10 regulation of the expression of Bcl-2 in IL-6/sIL-6Rα–induced RA FLS

**Figure 5:**
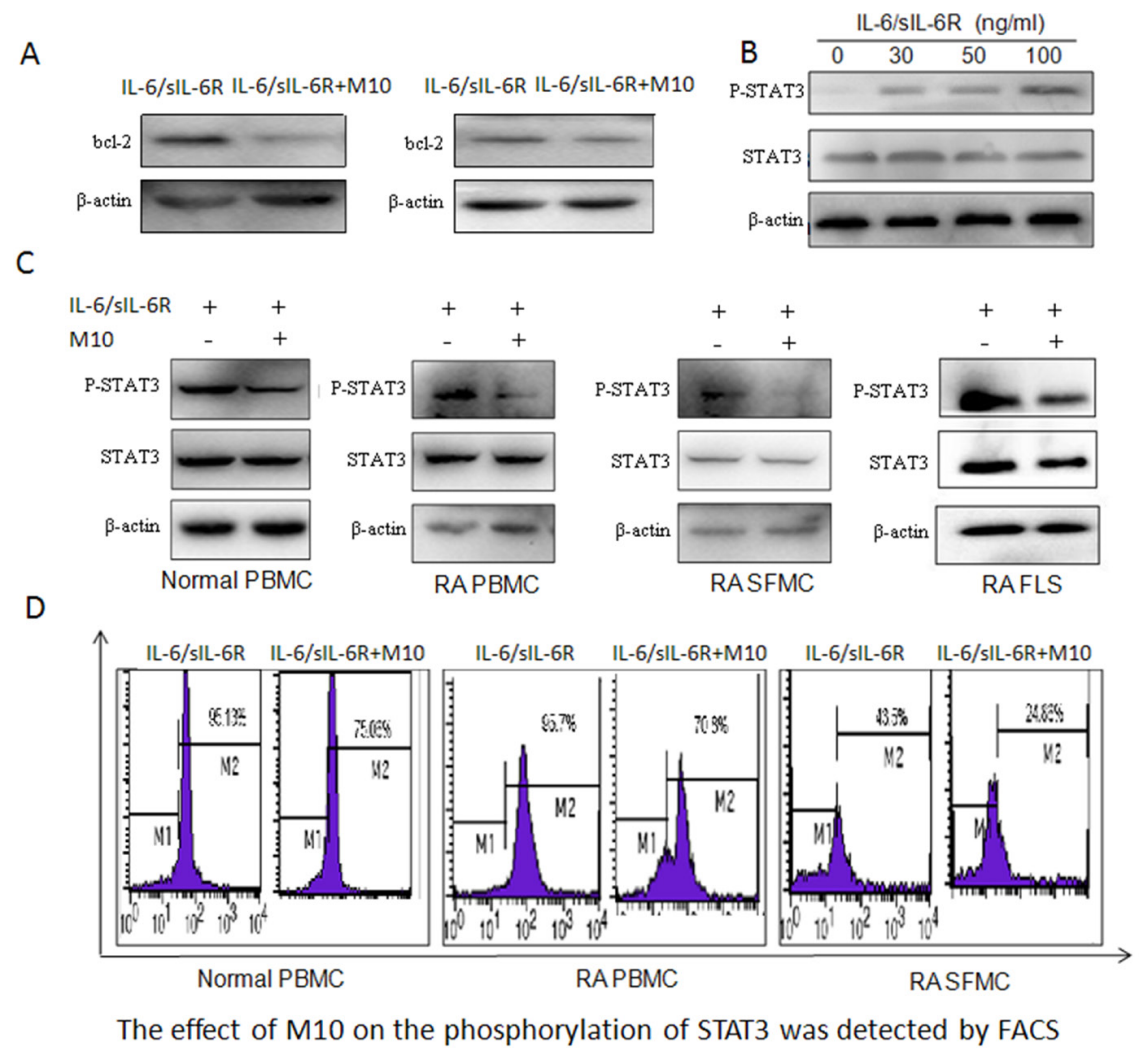
M10 intervention of the IL-6 signaling pathway in RA FLS **(A)** Western blotting determination of Bcl-2 levels in RA PBMC and FLS pretreated with M10 and cultured with IL-6/sIL-6Rα (100 ng/mL) for 72 h. **(B)** Western blot detection of STAT3 phosphorylation in RA FLS following 3-day stimulation with IL-6 and sIL-6Rα. **(C)** Western blot detection of STAT3 phosphorylation in normal PBMC, RA PBMC, RA SFMC, and RA FLS pretreated with M10 and cultured with IL-6/sIL-6Rα (100 ng/mL) for 30 min. **(D)** FACS detection of the effect of M10 on STAT3 phosphorylation in normal PBMC, RA PBMC, and RA SFMC.

In RA pathogenesis, synovial tissue is hyperplasic. Abnormal changes of several members of the pro-apoptotic protein family, especially the Bcl-2 family are highly expressed, while levels of the pro-apoptotic factor Bax are increased slightly. The increase of Bcl-2, Bax and Bcl-xl formed two heterologous dimers, inhibited downstream apoptotic cascade, thereby affecting the activation of apoptotic protease, causing the imbalance between synovial cell proliferation / apoptosis, leading to excessive proliferation of synovial cells, aggravation of RA disease. JAK/STAT signaling pathway plays important roles in RA, some studies have also found that JAK/STAT signaling pathway blockers can significantly reduce the expression levels of bcl-2, Bcl-xl genes and proteins, indicating that Bcl-2 is an important target molecule of JAK/STAT signaling pathway. Here, we detected significantly increased Bcl-2 levels in RA SF (Figure 1H); M10 inhibited the expression of Bcl-2 in the PBMC and FLS of the patients with RA (Figure 5A).

### The function of M10 on the STAT3 signaling pathway

We all know STAT3 plays a key role in the process of RA disease. It is closely related to bone destruction, cell proliferation, and apoptosis, at the same time, it is critical for controlling osteoclastogenesis by activating gp130-mediated cytokines so it has been speculated that it may be responsible for M10 downregulation of RANKL. We studied the main pathways triggered by IL-6/sIL-6Rα, specifically, that for STAT3 activation. RA FLS were exposed to different concentrations of IL-6/sIL-6Rα for 20 min, and phosphorylated STAT3 levels increased in tandem with the IL-6/sIL-6Rα concentrations (Figure 5B). Then, we estimated the influence of M10 on STAT3 activation levels in normal PBMC, RA PBMC, RA SF mononuclear cells (SFMC), and RA FLS. We treated the cells with 50 μg/mL M10 for 2 h before being treated with 100 ng/mL IL-6/sIL-6Rα for 30 min. M10 significantly decreased STAT3 phosphorylation (Figure 5C, 5D).

## DISCUSSION

Local high expression of IL-6 plays an important role in joint destruction by promoting osteoclast maturation, formation, and activation of FLS, and synovial proliferation, culminating in joint damage in patients with RA [13]. In the present study, high expression of IL-6 and sIL-6Rα and low expression of the trans-signaling inhibitor sgp130 were detected in the SF and serum of patients with RA. We also found gp130 upregulation in RA PBMC; at the same time, gp130 expression in the osteoclasts of patients with RA were higher than that in the controls. Overall, we demonstrate that patients with RA have elevated IL-6–gp130 cell signaling.

Formed by the fusion of mononuclear precursors of monocytes/macrophages, osteoclasts are ultimately responsible for the bone destruction in RA [13]. RANKL promotes the activation and differentiation of osteoclast and inhibits the apoptosis of osteoclast, leading to an increase resorption of bone. Factors such as IL-1, TNFα, IL-17, and IL-6 regulate RANKL expression and synthesis. Hashizume et al. demonstrated that IL-6 stimulated the expression of RANKL at the presence of sIL-6Rα in FLS; furthermore, only in association with sIL-6Rα, IL-17 and TNFα increased the expression of RANKL [5, 7, 13]. This suggests that IL-6 trans-signaling pathway activation could induce genesis of osteoclast through promoting the expression of RANKL in the RA FLS. WNT5A, a conserved target of the STAT3 signaling cascade, also plays a key role in RA pathogenesis. In this context, we show that IL-6-sIL-6Rα upregulates the expression of WNT5A and RANKL in synovial fibroblasts [14].

Many of the newer agents for treating RA suppress the main molecular pathways in the pathological process of RA. Biological drugs that target the cytokines related to the pathogenic signaling have leaded to revolutionary in the treatments of RA [15]. IL-6 induced bone resorption in osteoclasts, depending on the sufficient activation of gp130 signals. The characteristics of gp130 knockout mice are increased giant but insufficient osteoclasts, accompanied by decreased bone resorption and by defective osteoblasts with reduced RANKL expression after stimulation [16]. In the present study, M10 (50 μg/mL) inhibition of gp130 signaling suppressed the expression of RANKL and WNT5A in IL-6-sIL-6Rα–induced RA FLS. *In vivo*, M10 significantly alleviated joint swelling, alleviated arthritis and clinical scores in the CAIA mouse model.

**Figure 6:**
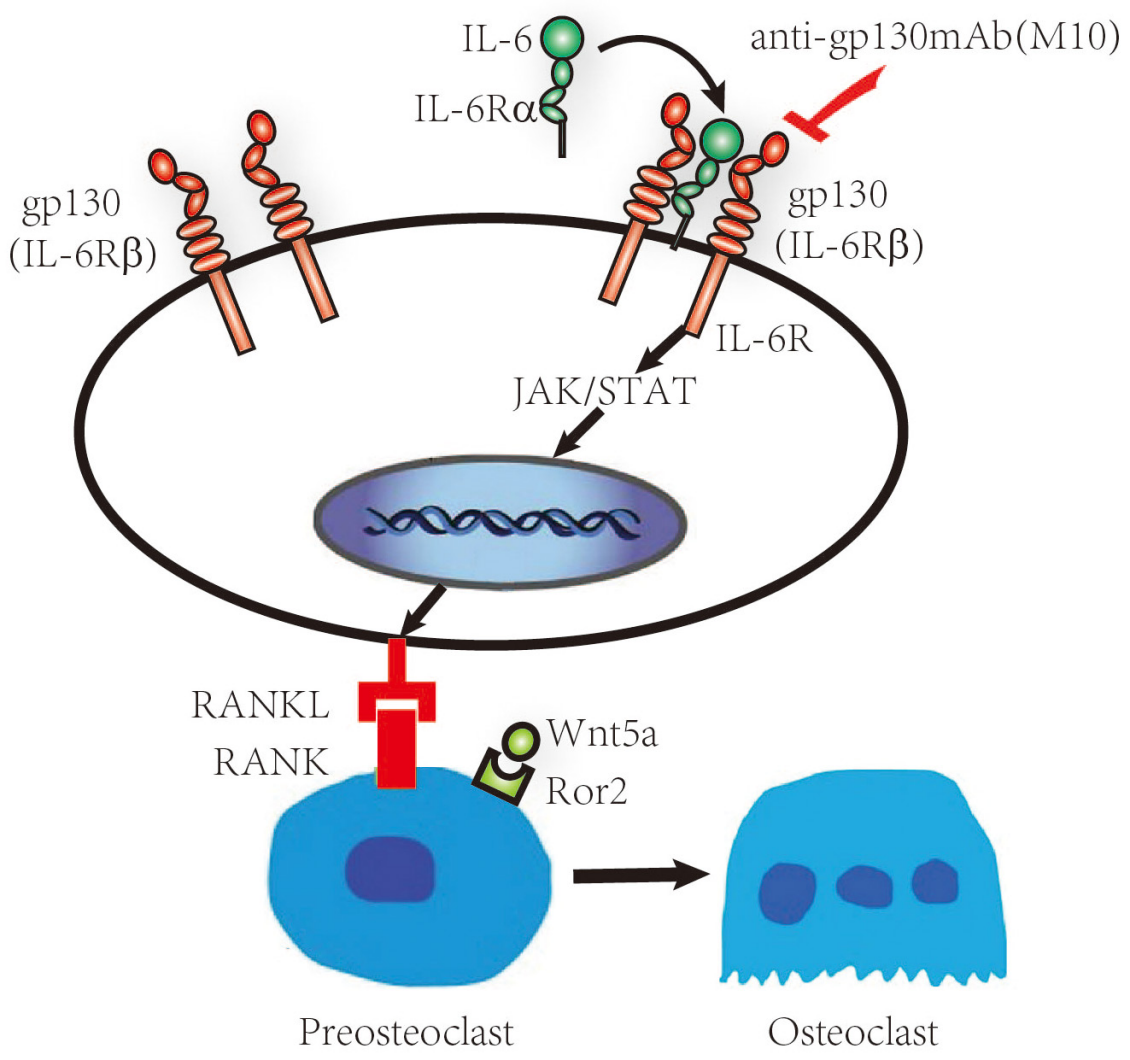
Schematic representation of the IL-6/sIL-6Rα/gp130 signaling pathways in RA FLS RA FLS release IL-6, which then interacts with IL-6Rα and gp130, forming a hexameric signaling complex and subsequently activating the JAK–STAT pathway and initiating RANKL, WNT5A, and Bcl-2 production.

IL-6 binding to sIL-6Rα activates the JAK and STAT3. STAT3 is an important molecule in the pathogenesis of RA. Stromal/osteoblastic cell line (UAMS-32) with dominant negative STAT3 protein treated with IL-6/sIL-6Rα, was failed to induce the expression of RANKL [17, 18]. Hashizume M et al found STAT inhibitor treated IL-6/sIL-6R-pretreated FLS decreased RANKL expression [7, 18]. In the STAT3 gene knockout mice, expression of RANKL was suppressed by treatment with IL-6 and IL-6R. Therefore STAT3 is important for controlling osteoclastogenesis by activating gp130-mediated cytokines [18]. We studied whether M10 inhibited the production of RANKL by reducing the activity of STAT3 in IL-6–sIL-6Rα treated FLS. The IL-6/sIL-6Rα leaded to increased STAT3 phosphorylation and increased the expression of RANKL protein in a dose- and time-dependent manner. In M10-treated cells, the improved *RANKL* mRNA expression and STAT3 phosphorylation were significantly lower than that in the cells not treated with M10. Therefore, M10 downregulates the expression of RANKL and WNT5A in IL-6/sIL-6Rα–pretreated FLS through the JAK–STAT3 signaling pathway as illustrated in Figure 6. These studies reveal that the protective effect of M10 in the destruction of bone is associated with the decreased RANKL and WNT5A production and STAT3 phosphorylation in FLS.

In summary, the IL-6–sIL-6Rα–gp130 complex plays a key role in the pathogenesis of RA through the action of synovitis and the destruction of bone. Accordingly, it is regarded as a promising target for treatment. In this research, we have developed an important antibody M10, which binds to gp130 with high affinity. M10 inhibited RANKL and WNT5A expression in RA synoviocytes both *in vivo* and *in vitro* by downregulating the JAK-STAT3 pathway; at the same time, it ameliorated the arthritis in the CAIA mouse model *in vivo*. In conclusion, we consider gp130 as a very important target molecule for the treatment of RA.

## MATERIALS AND METHODS

### Patients

Patients with RA or OA were enrolled in the study from 2008 to 2014 at the Shanghai Guanghua Hospital of Integrated Traditional Chinese and Western Medicine (Shanghai, China). All patients met the American College of Rheumatology criteria for RA and OA [19]. Each patient signed an informed consent form, and the hospital’s Human Research Ethics Committee approved the protocol.

### Animals

BALB/c mice (20–23 g, 8–10 weeks old) were purchased from the Shanghai Laboratory Animal Center, Chinese Academy of Sciences (Shanghai, China). Male A/J mice (6–8 weeks old, The Jackson Laboratory, Bar Harbor, ME, USA) were purchased from the Nanjing Experimental Model Animal Center, Nanjing University (Nanjing, China) as described previously [20]. The mice were housed according to institutional guidelines. The Ethics Committee of Laboratory Animals Welfare of Shanghai Jiao Tong University School of Medicine approved all animal procedures.

### Generation of anti-gp130 mAb

We immunized 10 A/J mice with gp130 antigen three times every 2 weeks. Before the final boost, we collected serum from a tail-bleed of each immunized mouse to detect the serum-specific antibody titer. The serum from all mice had high binding signals to the gp130 antigen. Spleen cells from the immunized mice were fused with mouse myeloma Sp2/0 cells to generate the antibody hybridoma. High-throughput screening was used to screen for stronger antigen binding from fusion cells/colonies. The hybridoma with the highest binding signals was selected for subcloning and antibody affinity purification.

Determination of the affinity constant of the mAb against human gp130 and binding kinetics was performed using Biacore X100(GE, USA), where we detected goat anti-mouse Fc antibodies on the chip, calculated the mAb concentration according to the formula provided by the manufacturer., and then proceeded according to the instrument indication.

### ELISA

Samples of peripheral blood and SF were collected from patients with RA, patients with OA, and healthy controls under aseptic conditions. Levels of IL-6, sIL-6Rα, sgp130, bcl-2, MMP3, TIMP and RANKL were assessed using a commercially available ELISA kit (R&D Systems, Minneapolis, MN, USA).

### Induction of CAIA mouse model and establishment of the treatment protocol

3 mice for each group are enrolled. The CAIA model was induced in BALB/c mice by injecting 2 mg anti-collagen antibody cocktail (Chondrex, Redmond, WA, USA) intravenously on day 0 and then treating the mice with 25 μg lipopolysaccharide (LPS) on day 3. The mice were monitored daily; disease severity was evaluated based on clinical signs of arthritis as reported previously [12]. Two blinded investigators performed the clinical evaluations, and the mean scores for inflammation and joint damage and for cartilage destruction were calculated. On day 3, i.e., after LPS treatment, the intervention group received 100 μg anti-gp130 mAb, while the control mice in which CAIA was not induced and non-intervention groups received phosphate-buffered saline (PBS).

### Histological staining and scoring

On day 12 after the induction of arthritis, the mouse knees and paws were harvested and fixed in 4% paraformaldehyde, decalcified, and paraffin-embedded. Serial sections were stained with HE (Sakura Finetek, Tokyo, Japan) and safranin O with fast green counterstain. Inflammation and joint damage were scored on a scale of 0 (no inflammation) to 3 (severe inflammation) based on the number of inflammatory cells. Cartilage destruction was scored on a scale of 0 (no loss) to 3 (complete loss of articular cartilage). Two blinded investigators performed the scoring, and the mean scores were calculated.

### Osteoclast differentiation

PBMC cells from the controls and from patients with RA were incubated in α-minimum essential medium (α-MEM, Invitrogen, USA) supplemented with 10% fetal bovine serum (FBS) (Defined, HyClone) and 20 ng/mL human GM-CSF (PeproTech) for 3 days to generate osteoclast precursors. The osteoclast precursors were incubated with 20 ng/mL GM-CSF and 40 ng/mL RANKL (PeproTech) for an additional 7 days. Cytokines were replenished every 3 days.

### Human FLS isolation and culture

The collected synovial tissues were minced and digested with 1 mg/mL collagenase type 1 (Invitrogen, Life Technologies, Nærum, Denmark) and 1% penicillin/streptomycin in Dulbecco’s modified Eagle’s medium (DMEM, high- glucose Invitrogen, USA) for 2 h at 37°C; the cells were collected after centrifugation and cultured in DMEM containing 10% FBS and 1% penicillin/streptomycin in 5% CO_2_ at 37°C. The culture medium was aspirated after 24 h to remove non-adherent synoviocytes and was changed every 48 h until the synoviocytes reached confluence. Then, the synoviocytes were passaged using 0.25% trypsin/1 mM EDTA (Invitrogen). The cells were cryopreserved between passage 3–8, and cells from these passages were used for all experiments [21].

### RNA isolation, reverse transcription (RT), and real-time PCR

RNA was isolated from the cell cultures using TRIzol (Invitrogen, Carlsbad, CA, USA) according to the manufacturer’s protocol. RNA (1 μg) was reverse-transcribed using a RT kit (Promega, Madison, WI, USA) and subsequently used for SYBR Green–based real-time PCR using a standard protocol as described previously [13]. The human primer sequences (Sangon Biotech, Shanghai, China) used for the RT-PCR were as follows: *IL6*, 5′-CAAGACATGCCAAAGTGCTG-3′ (sense) and 5′-TTGAGACTCATGGGAAAATCC-3′ (anti-sense); *RANKL*, 5′-ACCAGCATCAAAATCCCAAG-3′ (sense) and 5′-CCCCAAAGTATGTTGCATCC-3′ (anti-sense); *WNT5A*, 5′-ATTCTTGGTGGTGGTCGCTAGGTA-3′ (sense) and 5′-CGCCTTCTCCGATGTACTGC-3′ (anti-sense); and glyceraldehyde-3-phosphate dehydrogenase (*GAPDH*), 5′-GAAGGTCGGAGTCAACGGAT-3′ (sense) and 5′-CCTGGAAGATGGTGATGGG-3′ (anti-sense). The PCR conditions were 95°C for 10 minutes followed by 40 cycles at 95°C for 10 s, 56°C for 10 s, and 72°C for 30 s. The melting curve was assessed in the following program: 60°C for 1 min and 95°C continuous. The results were calculated using the comparative threshold cycle (ΔΔCt) method and are presented as fold increase relative to *GAPDH*.

### Immunohistochemistry

FLS were plated on cover glass and cultured with 100 ng/mL IL-6/sIL-6Rα with or without 50 μg/mL M10 for 3 days. Then, the cells were fixed with 4% buffered paraformaldehyde after washing with PBS, and then permeabilized with 0.5% Triton X-100 for 10 min at room temperature. The subsequent steps were performed as described previously [21]. The samples were incubated overnight at 4°C with antibodies against WNT5A (1:100, Abcam, Cambridge, MA, USA) and RANKL (1:100, R&D Systems).

### Western blotting

After cells or bone samples had been lysed, the protein concentrations were measured using a bicinchoninic acid protein assay kit. The lysates were centrifuged and denatured for 5 min at 94°C. Each protein sample underwent 10% sodium dodecyl sulfate–polyacrylamide gel electrophoresis and was transferred onto 0.45-μm polyvinylidene fluoride membranes (Millipore, Bedford, MA, USA). The membranes were blocked with 5% non-fat dry milk in Tris-buffered saline with Tween 20 (TBST) for 1 h, and probed with primary antibodies against RANKL (1:1000, R&D Systems), bcl-2 (1:1000, Abcam), WNT5A (1:1000, Abcam), or β-actin (1:5000, Abcam) at 4°C overnight. The membranes were then washed three times with TBST and incubated for 1 h with horseradish peroxidase–conjugated secondary antibodies (1:5000, Abcam). The proteins were visualized using an enhanced chemiluminescence detection system as recommended by the manufacturer.

### FACS to detect the phosphorylation of STAT3

The phosphorylation of STAT3 in normal PBMC, RA PBMC and RA SFMC was determined by flow cytometry. 1×10^6^ treated cells were washed by PBS/1% BSA twice. Added 250 μL BD Cytofix/Cytoperm buffer, waiting 30 minutes, washing one time by 1x washing buffer. After the addition of diluted Phospho-STAT3(Tyr705 Rabbit mAb), the cells were incubated for 60 min on ice, washing one time using 1x washing buffer. Cells were incubated with an FITC-labeled secondary antibody in 1x washing buffer for 60 min at 4°C. Cells were washed as previously described and resuspended. Fluorescence intensities were determined by a FACS Calibur flow cytometer (BD Biosciences).
